# Solution‐Processable, Ladder‐Branched Polyimides of Intrinsic Microporosity by [4+4] Cycloaddition for Membrane Gas Separation

**DOI:** 10.1002/adma.202513892

**Published:** 2025-10-15

**Authors:** Tae Hoon Lee, Pablo A. Dean, Jing Ying Yeo, Zachary P. Smith

**Affiliations:** ^1^ Department of Chemical Engineering Massachusetts Institute of Technology Cambridge Massachusetts 02139 United States; ^2^ Department of Future Energy Engineering Sungkyunkwan University Suwon 16419 Republic of Korea

**Keywords:** anthracene, gas separation, membranes, microporous polymers, post‐synthetic modification

## Abstract

Advancements in membrane‐based gas separation have the potential to address global challenges related to energy and the environment. However, new membrane materials must have excellent separation performance, stability, and processability, and simultaneously achieving all three metrics is extremely challenging. To circumvent these issues, a post‐synthetic modification of polyimides of intrinsic microporosity (PIM‐PIs) synthesized with a UV light (UV)‐reactive anthracene co‐monomer is reported. UV irradiation on the PIM‐PI solution converts the anthracene units into dianthracene linkages by [4+4] cycloaddition, while the resultant PIM‐PI is still solution‐processable due to the branched structure. The ladder‐like dianthracene moieties significantly increased both microporosity (<20 Å) and ultramicroporosity (<7 Å) of the precursor PIM‐PI. Notably, the UV‐treated PIM‐PI membrane exhibits a large boost in pure‐gas CO_2_ permeability by up to 260%, reaching 376 barrer, while maintaining CO_2_/CH_4_ ideal selectivity of 35 at 1 bar. Moreover, the developed membrane material has enhanced stability against physical aging and plasticization and showcases excellent CO_2_/CH_4_ mixed‐gas selectivity (>30 up to 31 bar feed pressure), which surpasses the 2018 mixed‐gas upper bound.

## Graphical abstract

An effective post‐synthetic modification strategy utilizing [4+4] cycloaddition is demonstrated to fabricate ladder‐branched polyimides of intrinsic microporosity (PIM‐PIs) with significantly enhanced performance characteristics for membrane‐based gas separation.

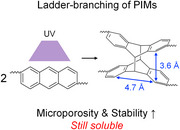

## 1 Introduction

Industrial chemical separations account for 10–15% of total global energy consumption.^[^ ^1^ ^]^ This reality has led to an urgent demand for energy‐efficient separation technologies to reduce costs and mitigate emissions that contribute to climate change.^[^ ^1^, ^2^, ^3^ ^]^ Membrane technology is a promising alternative to conventional processes (e.g., distillation and absorption) due to its high energy efficiency, operational simplicity, and small footprint.^[^ ^4^, ^5^ ^]^ In particular, membrane‐based gas separation offers promising opportunities for the decarbonization of the power and industrial sectors through integration in hydrogen production, fossil fuel power plants, and natural gas systems.^[^ ^5^, ^6^ ^]^ For these applications, a polymer membrane material should be i) capable of allowing fast and selective transport of desired gas molecules, ii) durable against penetrant‐induced plasticization (i.e., stability in industrially relevant conditions with condensable gases) and physical aging (i.e., long‐term stability), and iii) solution‐processable for practical use as thin‐film composites or modularized hollow fiber membranes.^[^ ^4^, ^5^, ^6^ ^]^ Unfortunately, despite the tremendous research efforts on membrane materials over a span of decades, only a few polymers have been successfully deployed as commercial gas separation membranes (e.g., Matrimid, cellulose acetate (CA), and polysulfone (PSf)).^[^ ^5^, ^6^ ^]^

Ladder polymer networks are an emerging class of organic polymer materials, consisting of regularly repeating rings connected to each other through multiple shared edges, which enables them to display excellent size‐sieving capabilities.^[^ ^7^ ^]^ However, these materials often lack solution processability for membrane formation.^[^ ^8^, ^9^ ^]^ On the other hand, soluble polymers of intrinsic microporosity (PIMs) have been proposed as next‐generation membrane materials for gas separation.^[^ ^10^, ^11^, ^12^ ^]^ The abundant micropores (<20 Å) and ultramicropores (<7 Å) in PIMs are responsible for their excellent gas separation performance,^[^ ^13^, ^14^ ^]^ redefining the permeability–selectivity upper bounds for separations of several gas pairs in 2008,^[^ ^15^ ^]^ 2015,^[^ ^16^ ^]^ and 2019^[^ ^17^ ^]^ from the upper bounds originally reported in 1991.^[^ ^18^ ^]^ To date, numerous studies have focused on the synthesis of novel PIMs by directly incorporating a rigid and contorted monomer into the backbone (e.g., spirobisindane,^[^ ^10^ ^]^ spirobifluorene,^[^ ^19^ ^]^ Tröger's base,^[^ ^20^ ^]^ and iptycenes^[^ ^21^ ^]^). A variety of new polymerization chemistries have also been developed (e.g., ring‐opening metathesis polymerization (ROMP),^[^ ^22^ ^]^ palladium‐catalyzed polycondensation,^[^ ^23^ ^]^ and catalytic arene‐norbornene annulation (CANAL) polymerization^[^ ^24^ ^]^) to construct micropore‐generating side chains or unique backbone structures. However, these synthetic approaches are often laborious and time‐consuming due to the harsh conditions required for monomer synthesis, polymerization, and purification, which may also add costs to the final products.^[^ ^13^, ^17^, ^25^ ^]^ Moreover, under industrially relevant mixed‐gas conditions, traditional PIM materials often exhibit a substantial drop in the separation performance compared with that measured under pure‐gas conditions due to penetrant‐induced plasticization.^[^ ^25^, ^26^, ^27^, ^28^ ^]^ This behavior is commonly benchmarked by an experimental mixed‐gas upper bound for CO_2_/CH_4_ separation reported in 2018,^[^ ^25^ ^]^ which is defined by an underperforming upper bound compared to that of the 2019 pure‐gas upper bound.^[^ ^17^ ^]^ Lastly, intensified physical aging in high‐free‐volume PIMs is another critical challenge for their long‐term stability due to the densification of polymer matrices as time elapses, resulting in a continuous decrease in gas permeability.^[^ ^24^, ^26^, ^29^, ^30^ ^]^

Post‐synthetic modification of existing PIMs is a potential solution to circumvent these issues, since this approach is relatively facile compared to the discovery of new synthetic routes.^[^ ^30^, ^31^, ^32^, ^33^ ^]^ For example, chemical functionalization has been primarily explored for PIM‐1, the archetypal PIM,^[^ ^25^ ^]^ by converting its nitrile groups into different functional groups such as carboxylic acid,^[^ ^34^ ^]^ amine,^[^ ^30^, ^33^ ^]^ amidoxime,^[^ ^35^ ^]^ and tetrazole.^[^ ^36^ ^]^ Crosslinking PIMs by thermal or chemical treatment is another common strategy.^[^ ^33^, ^37^, ^38^, ^39^ ^]^ In general, these post‐synthetic modifications densify the polymer matrix by inducing hydrogen bonding or physical/chemical crosslinking between the polymer chains, which contribute to either a selectivity boost or better physical or plasticization stability in the resultant PIM membranes.^[^ ^33^, ^34^, ^38^, ^39^ ^]^ However, these benefits often come at the cost of enhanced excessive intermolecular forces, resulting in decreased gas permeability, and frequently, insoluble PIMs after the modifications.^[^ ^30^, ^33^, ^34^ ^]^ The above examples demonstrate that leveraging all three essential features – i) separation performance, ii) stability, and iii) processability – has been extremely challenging in developing advanced membrane materials for gas separation applications.

Branching, which is an alternative to the typical crosslinking strategy, refers to the attachment of additional chains to the main backbone of a polymer without reaching a gel point that would result in a network structure. Branching can improve the thermal and chemical stabilities of membranes while simultaneously maintaining good solubility.^[^ ^40^, ^41^ ^]^ For example, Zhang *et al.* recently reported a design of branched poly(aryl piperidinium) membranes for redox flow batteries by using rigid and contorted spirobifluorene monomers to control their microporosity without losing their solubility.^[^ ^41^ ^]^ Our group also recently reported a solution‐processable branched poly(arylene ether) (PAE), which showed excellent gas separation performance as well as remarkable plasticization resistance.^[^ ^23^ ^]^ We envision that if post‐synthetic modification can form a branched structure connected by ladder‐like linkages, the resultant polymer will overcome the permeability–selectivity trade‐off and stability issues in gas separation membranes while still featuring the solution processability of the nascent membrane film.

Polyimides of intrinsic microporosity (PIM‐PIs) are a subclass of PIMs, in which bulky or contorted moieties (i.e., PIM units) are incorporated into rigid polyimide backbones to generate large free volume elements and high surface area.^[^ ^25^ ^]^ Owing to the versatile chemistry of imide formation, which allows for a broad choice of diamine and dianhydride building blocks, and the excellent solution processability intrinsic to polyimides, PIM‐PIs uniquely combine structural tunability, robust thermal and mechanical stability, and high permeability, establishing them as one of the important and adaptable families of high‐performance membrane materials.^[^ ^21^, ^25^, ^43^ ^]^ To this end, we report a rational design of PIM‐PIs synthesized with a photo‐reactive anthracene monomer followed by a post‐synthetic modification via [4+4] cycloaddition to form ladder‐branched PIM‐PI membranes. Detailed characterization of the developed PIM‐PIs confirms their ladder‐branched structure coupled with enhanced microporosity, resulting in excellent gas separation performance as well as enhanced stability.

## 2 Results and Discussion

**Figure 1:**
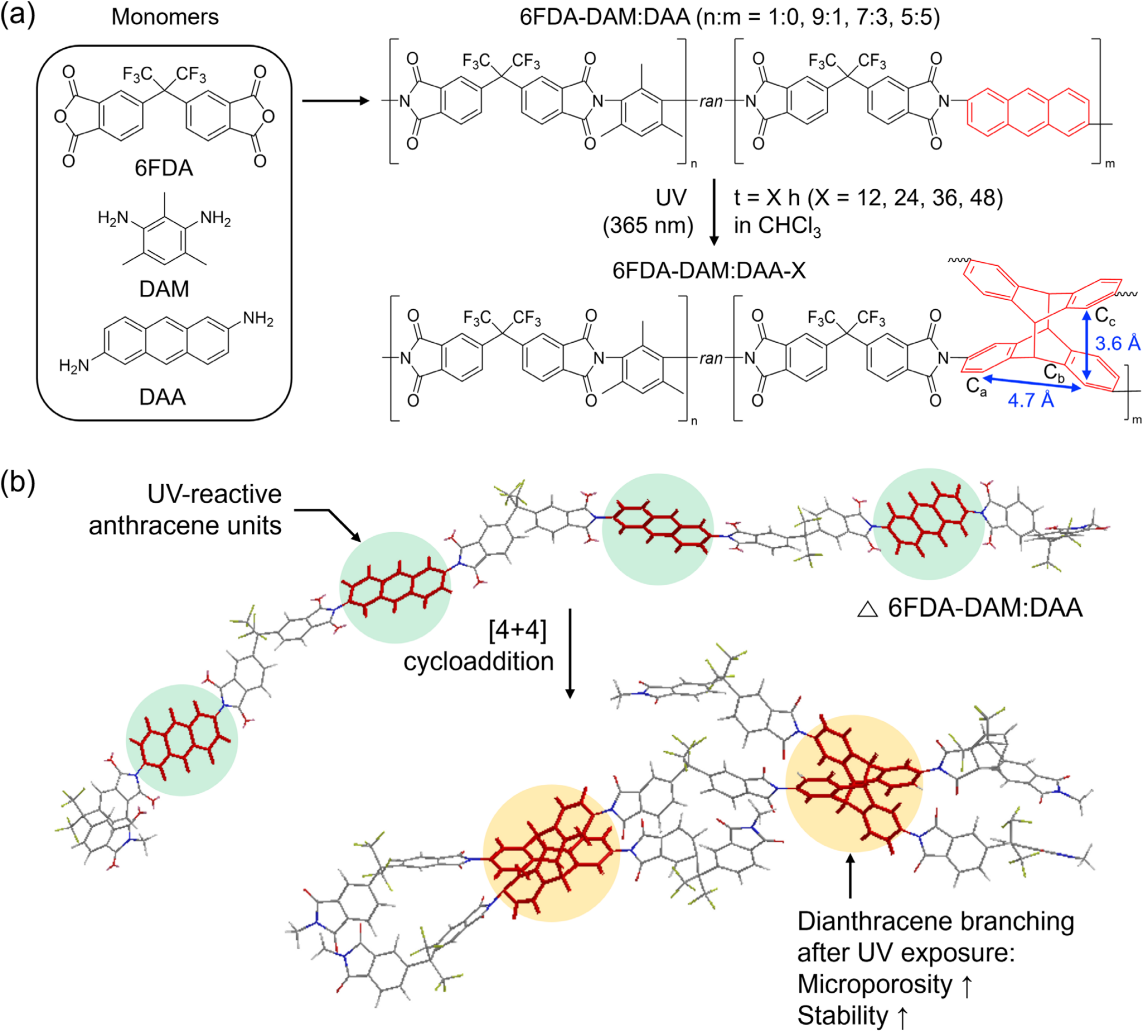
Schematic illustration. a) Synthesis of dianthracene‐containing copolyimides (6FDA‐DAM:DAA‐X) by ultraviolet (UV)‐light‐induced [4+4] cycloaddition (6FDA: 4,4′‐(hexafluoroisopropylidene) diphthalic anhydride, DAM: 2,4‐diaminomesitylene, and DAA: 2,6‐diaminoanthracene). *ran* indicates that the copolyimides are random copolymers. The distances between two carbon pairs (two adjunct C_a_ carbons (4.7 Å) and two adjunct C_c_ carbons (3.6 Å)) are reproduced from ref. [50]. The structure of 6FDA‐DAM:DAA‐X assumes syn‐dianthracene units. b) Hypothesized 3D equilibrated structures of 6FDA‐DAM:DAA and 6FDA‐DAM:DAA‐X copolyimides generated using the Chem3D Pro 12.0 software package. The structures assume DAM:DAA (n:m) ratio of 0:1, 100% conversion of anthracene units, and syn‐dianthracene units.

4,4′‐(Hexafluoroisopropylidene) diphthalic anhydride (6FDA) has been widely used to prepare high‐free‐volume polyimides for gas separations because it contains rigid and bulky ─C(CF_3_)_2_ groups that offer lower chain packing density and increased fractional free volume (FFV).^[^ ^42^ ^]^ Likewise, 2,4‐diaminomesitylene (DAM) is a bulky diamine that can further enhance FFV due to the presence of three ─CH_3_ groups.^[^ ^43^ ^]^ As a result, 6FDA‐DAM polyimide shows a high FFV (0.25) and Brunauer–Emmett–Teller (BET) surface area (400–500 m^2^ g^−1^), which classifies it as a PIM‐PI.^[^ ^29^, ^43^ ^]^ As an alternative approach, anthracene can potentially be used to enhance FFV by ladder‐branched structures if dimerized with neighboring anthracenes using UV light.^[^ ^44^, ^45^, ^46^, ^47^ ^]^ Based on the above rationales, we chose 6FDA‐DAM as a representative PIM‐PI and synthesized UV‐reactive copolyimides by using 2,6‐diaminoanthracene (DAA) as a co‐diamine monomer with different DAM:DAA ratios (i.e., 6FDA‐DAM:DAA, **Figure** **1**a). UV irradiation with wavelengths >350 nm is expected to post‐synthetically convert the anthracene units into dianthracene by [4+4] cycloaddition.^[^ ^44^ ^]^ Notably, the resultant dianthracene linkage is an analog to micropore‐generating ladder‐like pentiptycenes that feature internal free volume (IFV). Interestingly, these units have been explored as a building block to prepare PIMs.^[^ ^48^, ^49^ ^]^ The size of IFV in dianthracene ranges from 3.6 to 4.7 Å,^[^ ^50^ ^]^ which may contribute to improving the size‐sieving ability of the resultant membranes. Figure 1b shows the hypothetical 3D structure of the dianthracene‐containing copolyimides prepared by X = 12, 24, 36, and 48 h of UV exposure to the copolyimide precursor (6FDA‐DAM:DAA‐X), where X designates the hours of UV exposure in solution.

6FDA‐DAM:DAA copolyimides were synthesized in one‐pot, two‐step reaction: i) to form poly(amic acid) and ii) to form polyimide through solution‐state chemical imidization (Table S1, Supporting Information).^[^ ^51^ ^]^^1^H nuclear magnetic resonance (NMR) and Fourier‐transform infrared spectroscopy (FT‐IR) spectra confirm the incorporation of anthracene into 6FDA‐DAM:DAA, and peak intensities associated with anthracene become stronger when the molar ratio of DAA to DAM is increased (Figures S1 and S2, Supporting Information). Compared to the control 6FDA‐DAM (i.e., DAM:DAA = 1:0), 6FDA‐DAM:DAA exhibited a weight loss starting from ≈300 °C as shown by thermogravimetric (TGA) analyses, which is consistent with the mass loss region of DAA monomer (Figure S3, Supporting Information). The number average molecular weight (M_n_) of 6FDA‐DAM:DAA ranged from 23 000 to 99 000 g mol^−1^ as confirmed by gel permeation chromatography (GPC), which was high enough to cast a dense membrane film (Figure S4 and Table S2, Supporting Information). N_2_ and CO_2_ sorption analyses were used to evaluate the microporosity (>7 Å) and ultramicroporosity (<7 Å) of 6FDA‐DAM:DAA powders, respectively (Figures S5 and S6, Supporting Information). When the DAM:DAA ratio of 6FDA‐DAM:DAA decreased from 1:0 to 5:5, the N_2_‐based BET surface area decreased from 489 to 356 m^2^ g^−1^ (Table S3, Supporting Information). This reduction is ascribed to the lower concentration of the bulky DAM monomer and the enhanced chain packing efficiency by π–π stacking of the anthracene units.^[^ ^43^ ^]^ Isosteric heats of adsorption were calculated from the CO_2_ sorption isotherms at 273 and 298 K, which showed no significant variation among the samples (<7 kJ mol^−1^) (Figure S7, Supporting Information).^[^ ^52^ ^]^ Except for 6FDA‐DAM, which contains no DAA, all copolyimides exhibit strong UV peaks at 347, 365, and 385 nm, which are assigned to the characteristic π–π* transition bands of the anthracene (Figure S8, Supporting Information).^[^ ^45^, ^46^, ^47^ ^]^ This characterization indicates the UV‐reactive nature of 6FDA‐DAM:DAA copolyimides. In the following sections, the highest molar content of DAA (i.e., DAM:DAA = 5:5) was primarily investigated unless otherwise specified, given that it is expected to show the largest changes in the structural properties after UV treatment compared to 6FDA‐DAM.

**Figure 2:**
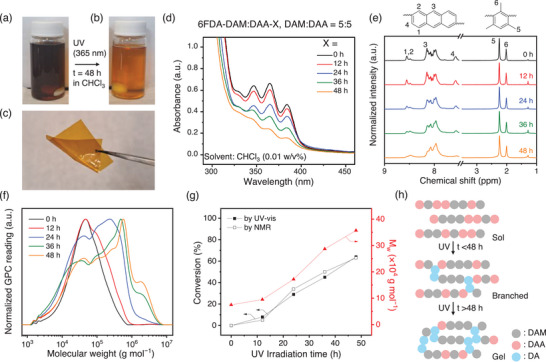
Characterization of 6FDA‐DAM:DAA‐X copolyimides (DAM:DAA = 5:5). Photo images of a) 6FDA‐DAM:DAA and b) 6FDA‐DAM:DAA‐48 solutions (1 w/v% in CHCl_3_). c) Photo image of a freestanding 6FDA‐DAM:DAA‐48 film (thickness = 50 ± 10 µm), showcasing its mechanical robustness. d) UV–vis absorbance spectra, e) ^1^H nuclear magnetic resonance (NMR) spectra (solvent: CDCl_3_), and f) gel permeation chromatography (GPC) curves of 6FDA‐DAM:DAA‐X exposed to 365 nm UV light for different irradiation times. g) Conversion of anthracene units calculated by UV–vis and NMR analyses and weight average molecular weight (M_w_) before (X = 0) to after UV irradiation of 6FDA‐DAM:DAA‐X for different irradiation times (X = 12, 24, 36, and 48 h). h) Schematic illustration of sol, partially crosslinked (i.e., branched), and gel states of 6FDA‐DAM:DAA‐X for different UV irradiation times (Note: DA represents dianthracene).

6FDA‐DAM:DAA‐X copolyimides containing dianthracene units were synthesized by irradiating 365 nm UV light to a 6FDA‐DAM:DAA solution in chloroform (CHCl_3_) with varying DAM:DAA molar ratios (1:0, 9:1, 7:3, and 5:5) at room temperature (Figure S9, Supporting Information). The color of the precursor solution was dark brown (**Figure** **2**a), which gradually turned into light brown as time elapsed (Figure 2b). Of note, gel formation was observed when the UV irradiation time exceeded 48 h (Figure S10, Supporting Information), indicating that the polymer became insoluble and could no longer be processed into films. Thus, 48 h was chosen as the maximum irradiation time. The resultant solution was used to cast 6FDA‐DAM:DAA‐X membranes by slow evaporation of solvent in a Teflon dish. The prepared films were mechanically robust and bendable (Figure 2c). Additional details on film fabrication are described in the Supporting Information.

UV‐vis spectra of 6FDA‐DAM:DAA‐X confirm that the intensity of peaks assigned to anthracene (347, 365, and 385 nm)^[^ ^45^, ^46^, ^47^ ^]^ became less pronounced by increasing the irradiation time (Figure 2d). In addition, ^1^H NMR spectra show that the intensities of the hydrogen peaks related to anthracene at 7.5–8.5 ppm significantly diminished after UV irradiation, while other characteristic peaks of the 6FDA‐DAM:DAA precursor (e.g., DAM: 2.0 and 2.2 ppm) remained largely unchanged (Figure 2e).^[^ ^47^ ^]^ The conversion of anthracene into dianthracene was calculated based on UV–vis and NMR spectra, which were in good agreement with each other, showing 64% and 63% conversion for 6FDA‐DAM:DAA‐48, respectively (Table S4, Supporting Information).^[^ ^45^, ^47^ ^]^ Also, the mass loss starting from 300 °C was much less noticeable for 6FDA‐DAM:DAA‐48 compared to 6FDA‐DAM:DAA (Figure S11, Supporting Information), proving its enhanced thermal stability. Likewise, tensile tests confirmed the enhanced mechanical robustness of 6FDA‐DAM:DAA‐48 compared to 6FDA‐DAM:DAA, which is attributed to increased chain entanglement and restricted chain mobility induced by the branched structure (Figure S12, Supporting Information). These results indicate the successful conversion of anthracene units in 6FDA‐DAM:DAA copolyimides into dianthracene by UV‐induced [4+4] cycloaddition.

Notably, the prepared 6FDA‐DAM:DAA‐X copolyimides were readily soluble, which enabled us to investigate their molecular weight distributions by GPC analysis. In addition, the solution processability of the 6FDA‐DAM:DAA‐X series is expected to be favorable for tuning their film morphology and preparing thin‐films.^[^ ^5^, ^6^ ^]^ As the irradiation time increased, the peak molecular weight shifted to higher values while the distribution became much broader (Figure 2f), indicating a higher weight average molecular weight (M_w_) and polydispersity index (PDI) (Table S5, Supporting Information). Similar molecular weight distributions have been observed in other branched polymer systems, such as for radical polymerization of acrylic monomers and ladder‐branched polyethylene.^[^ ^53^, ^54^ ^]^ Of note, in contrast to classic chain‐growth systems where branching can lead to a narrowing of the molecular weight distribution due to uniform growth kinetics,^[^ ^55^ ^]^ our system involves post‐synthetic branching of a backbone unit in pre‐formed linear polyimides, which fundamentally alters the probability for branching events. This post‐treatment introduces branching reactions heterogeneously across an existing population of polymer chains with varying lengths. As a result, some chains become significantly larger through multiple branching events or coupling, while others remain relatively unchanged.^[^ ^56^, ^57^ ^]^ This non‐uniform modification leads to a broadening of the molecular weight distribution and an increase in PDI, diverging from the behavior predicted for traditional branched polymerizations formed from multifunctional monomers.^[^ ^58^, ^59^ ^]^ Furthermore, some light chain scission may further broaden the PDI, as observed in Figure 2f. A strong correlation between the conversion and molecular weight also supports the branched structure of 6FDA‐DAM:DAA‐X (Figure 2g) as depicted in Figure 2h.^[^ ^53^, ^54^ ^]^

**Figure 3:**
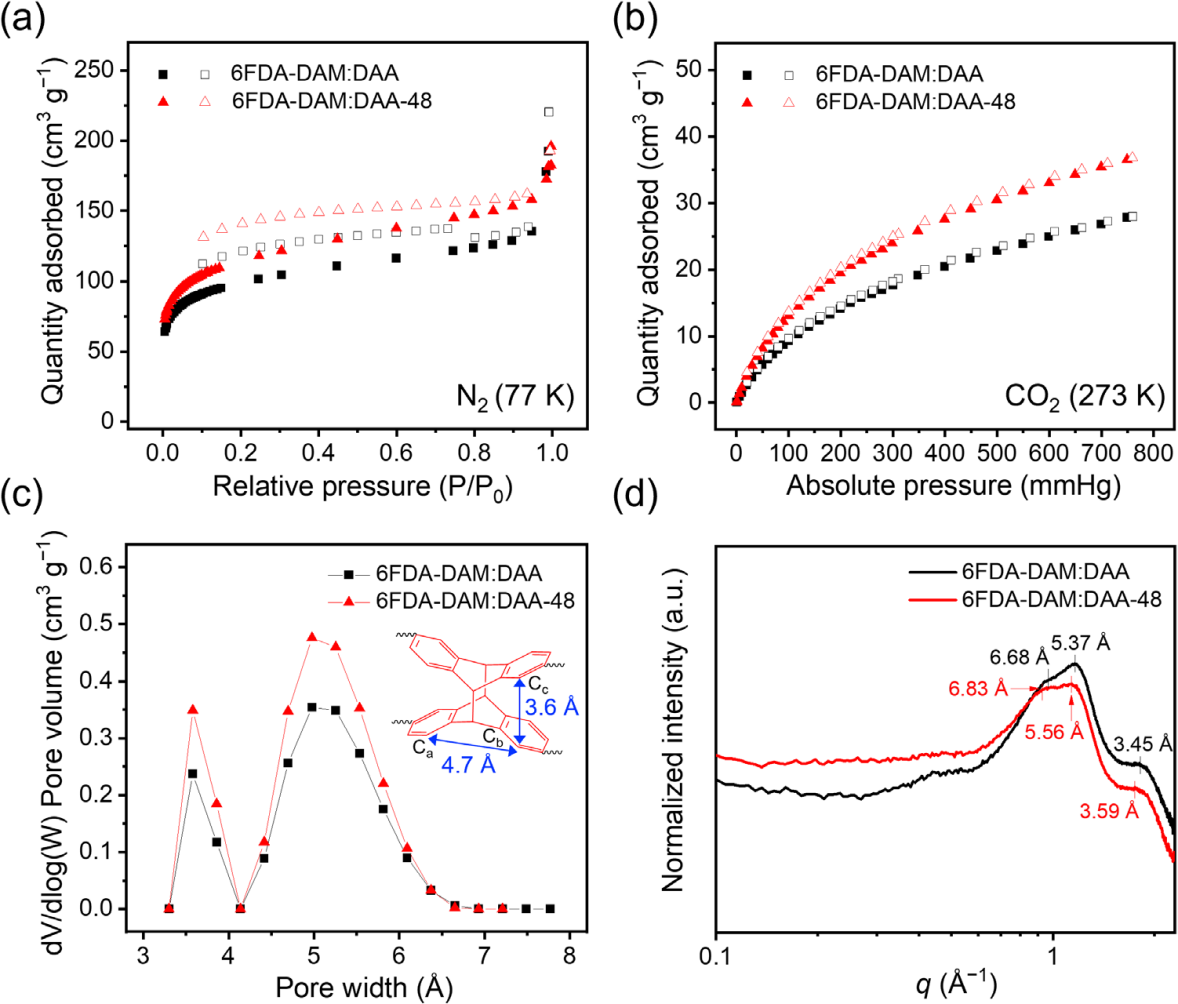
Microporosity analyses of 6FDA‐DAM:DAA and 6FDA‐DAM:DAA‐48 copolyimides (DAM:DAA = 5:5). a) N_2_ and b) CO_2_ adsorption–desorption isotherms measured up to 1 bar at 77 K and 273 K, respectively. Filled and unfilled symbols represent adsorption and desorption, respectively. c) Pore size distributions were determined using a non‐local density functional theory (NLDFT) model based on the data from (b). Inset image is reproduced from Figure 1a. d) Wide‐angle X‐ray scattering (WAXS) spectra. The numbers represent the *d*‐spacings corresponding to each peak.

The microporosity of the copolyimides was characterized by N_2_ adsorption–desorption analyses (**Figure** **3**a). Polymer powders were used for the porosity characterization to allow for fast diffusion of probe molecules for sorption.^[^ ^60^ ^]^ For 6FDA‐DAM:DAA‐48, the UV‐irradiated polymer solution was precipitated into methanol, a nonsolvent, to acquire powders for BET analysis. The BET surface areas of 6FDA‐DAM:DAA and 6FDA‐DAM:DAA‐48 were 356 and 453 m^2^ g^−1^, respectively. In addition to N_2_, CO_2_ has been acknowledged as a useful probe gas to investigate the ultramicroporous region (<7 Å) due to its smaller kinetic diameter (3.3 Å) compared to that of N_2_ (3.64 Å).^[^ ^61^, ^62^ ^]^ Langmuir surface areas of 6FDA‐DAM:DAA and 6FDA‐DAM:DAA‐48 were calculated from CO_2_ sorption isotherms at 273 K (Figure 3b), yielding 115 and 151 m^2^ g^−1^, respectively. These results correspond to 27% and 31% increases in specific surface area in microporous (based on N_2_ sorption isotherms) and ultramicroporous regions (based on CO_2_ sorption isotherms) (Table S6, Supporting Information).^[^ ^63^ ^]^ Of note, the deviation in isosteric heat of adsorption for CO_2_ (obtained by sorption isotherms at 273 and 298 K) between 6FDA‐DAM:DAA and 6FDA‐DAM:DAA‐48 is only marginal (<7 kJ mol^−1^), which allows us to safely exclude the effect of favorable binding between the sample and CO_2_ molecules (Figures S13 and S14, Supporting Information), which would convolute interpretability.^[^ ^63^ ^]^

The pore size distributions of the copolyimides were calculated from the CO_2_ sorption isotherms by the non‐local density functional theory (NLDFT) model (Figure 3c).^[^ ^63^ ^]^ Compared to 6FDA‐DAM:DAA, 6FDA‐DAM:DAA‐48 displayed an increase in the pore volumes of submicroporosity (3–4 Å) and ultramicroporosity (4–7 Å).^[^ ^63^ ^]^ This difference is attributed to the formation of the dianthracene unit, which possesses an IFV size ranging from 3.6 to 4.7 Å.^[^ ^50^ ^]^ Wide‐angle X‐ray scattering (WAXS) spectra (Figure 3d) indicate a decrease in the intensity at 1.82 Å^−1^ (*d*‐spacing = 3.45 Å) after UV irradiation, which is ascribed to the π–π stacking between anthracene units. This peak was also slightly shifted to 1.75 Å^−1^ (*d*‐spacing = 3.59 Å) for 6FDA‐DAM:DAA‐48, which closely correlates with the distance between two adjacent C_c_ carbons in dianthracene.^[^ ^50^ ^]^ Additionally, two other WAXS peaks at 0.94 and 1.17 Å^−1^ (*d*‐spacing = 6.68 and 5.37 Å) were shifted to smaller values (0.92 and 1.13 Å^−1^, *d*‐spacing = 6.83 and 5.56 Å) after UV irradiation of 6FDA‐DAM:DAA to 6FDA‐DAM:DAA‐48, which may be attributed to the increase in free volume within the ultramicroporous regime from the formation of the dianthracene unit. These results are consistent with the NLDFT‐based pore size distributions.

We also prepared and characterized 6FDA‐DAM:DAA‐X copolyimides with a DAM:DAA ratio of 9:1 and 7:3 to confirm the generalizability of our concept. Of note, 6FDA‐DAM:DAA with a lower concentration of DAA was lighter in color, which led to a better absorbance of UV light and thus facilitated the dimerization reaction. Therefore, we chose shorter irradiation times for these samples (X = 12 and 36 h for DAM:DAA = 9:1 and 7:3, respectively) to avoid gel formation. 6FDA‐DAM (without any DAA) was also irradiated with UV for 12 h to serve as a control. For all copolyimides, both UV‐vis and ^1^H NMR spectra confirm 60–79% of conversion of anthracene into dianthracene for 6FDA‐DAM:DAA‐X copolyimides, while no noticeable change was observed for 6FDA‐DAM after UV irradiation (Figures S15 and S16 and Table S7, Supporting Information). 6FDA‐DAM:DAA‐X also exhibited increases in M_w_ and PDI coupled with enhanced thermal stability (Figures S17 and S18 and Table S8, Supporting Information). After UV irradiation of the copolyimides, enhanced microporosity and ultramicroporosity were also confirmed by N_2_ and CO_2_ adsorption–desorption studies. In general, the extent of the increase in the specific surface area became more pronounced as the molar content of DAA increased (Figures S19–S22 and Table S9, Supporting Information), which is attributed to the higher concentration of micropore‐generating dianthracene units. These results indicate that, regardless of the DAM:DAA ratio, anthracene was successfully converted into dianthracene through UV irradiation, confirming the generalizability of this concept.

**Figure 4:**
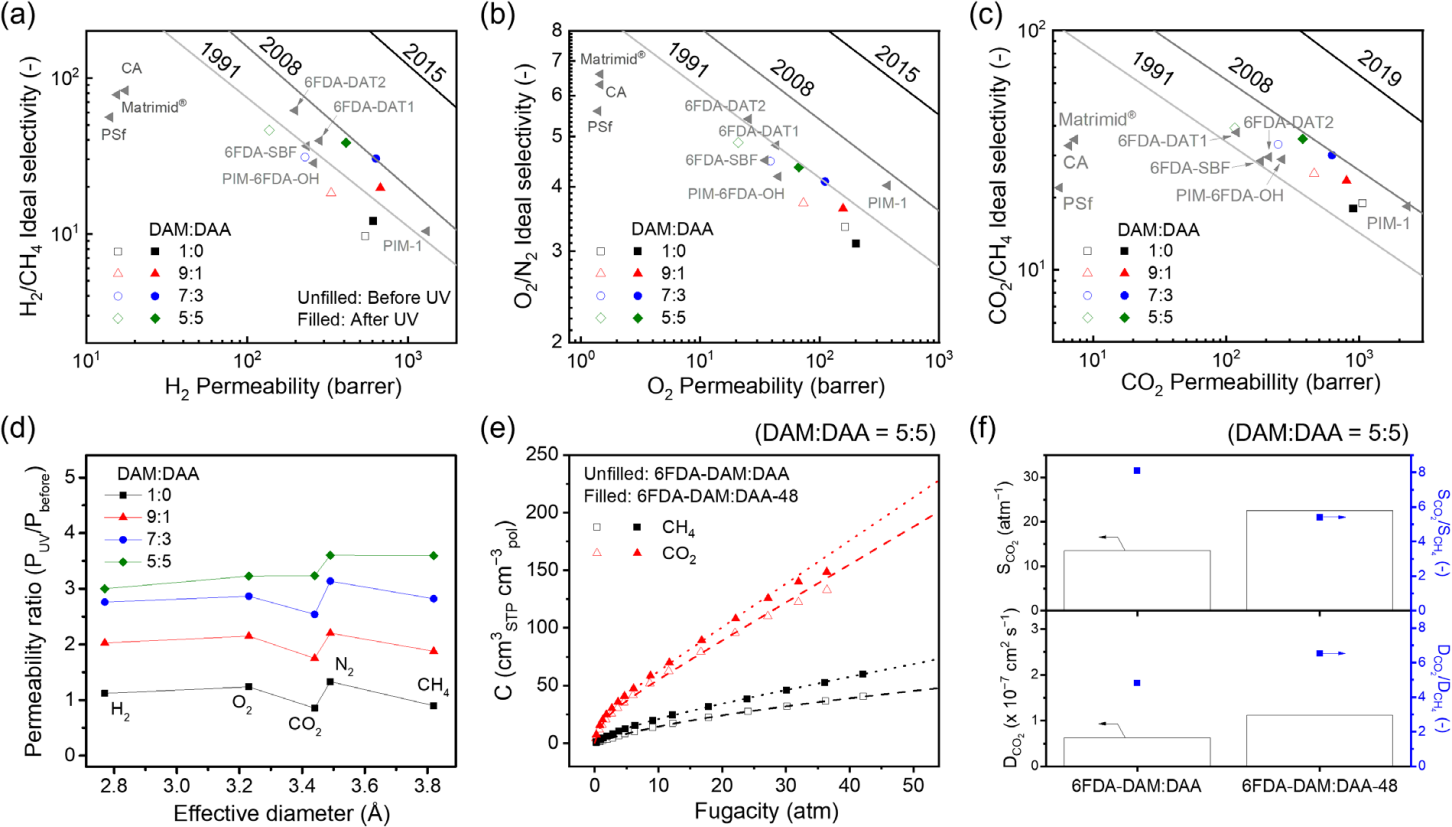
Pure‐gas separation performances of 6FDA‐DAM:DAA (unfilled symbols) and 6FDA‐DAM:DAA‐X (filled symbols) membranes plotted on upper bound plots for a) H_2_/CH_4_, b) O_2_/N_2_, and c) CO_2_/CH_4_ measured at 35 °C and 1 bar. Gray triangles indicate the gas separation performance of Matrimid, CA, and PSf (commercial membranes) and several PIM‐based membranes reported in refs. [25] and [43]. The numbers above the solid lines represent the published year of each upper bound in 1991,^[^ ^18^ ^]^ 2008,^[^ ^15^ ^]^ 2015,^[^ ^16^ ^]^ and 2019.^[^ ^17^ ^]^ d) Permeability ratio of UV‐treated samples (P_UV_) to untreated samples (P_before_) versus the effective diameter of gas molecules. X = 12 h for DAM:DAA = 1:0 and 9:1 while X = 36 h or 48 h for DAM:DAA = 7:3 and 5:5, respectively. e) Sorption isotherms of CH_4_ and CO_2_ for 6FDA‐DAM:DAA (unfilled symbols) and 6FDA‐DAM:DAA‐48 (filled symbols) membranes measured at 35 °C. Dotted and dashed lines represent the fitted results derived from the dual‐mode sorption model. f) Diffusion and sorption coefficients and selectivities ($D_{{\mathrm{CO}}_{2}}$/$D_{{\mathrm{CH}}_{4}}$ and $S_{{\mathrm{CO}}_{2}}$/$S_{{\mathrm{CH}}_{4}}$) of 6FDA‐DAM:DAA and 6FDA‐DAM:DAA‐48 membranes. Note that DAM:DAA ratio is 5:5 for (e) and (f). [Correction added on October 20, 2025, after first online publication: Caption of Figure 4f has been updated.]

Next, pure‐gas (H_2_, O_2_, CO_2_, N_2_, and CH_4_) transport properties of 6FDA‐DAM:DAA and 6FDA‐DAM:DAA‐X (X = 12, 36, and 48 for DAM:DAA = 9:1, 7:3, and 5:5, respectively) membranes were examined at 1 bar and 35 °C. For several industrially relevant gas pairs (e.g., H_2_/CH_4_, O_2_/N_2_, and CO_2_/CH_4_), increasing the molar content of DAA in 6FDA‐DAM:DAA resulted in an increase in selectivity coupled with a loss in gas permeability, likely due to the reduced free volume (Table S10, Supporting Information). On the other hand, for all gases, a significant increase in gas permeability was observed for 6FDA‐DAM:DAA‐X membranes compared to each 6FDA‐DAM:DAA precursor. Increasing the DAA concentration in the 6FDA‐DAM:DAA precursor also resulted in a more pronounced improvement in gas permeability after UV irradiation. More importantly, the selectivity loss was only marginal for 6FDA‐DAM:DAA‐X membranes compared to the permeability increase, placing these membranes close to the 2008 Robeson upper bound for H_2_/CH_4_, O_2_/N_2_, and CO_2_/CH_4_ separations (**Figure** **4**a–c). For example, in the case of the CO_2_/CH_4_ pair, the CO_2_ permeability increased from 116 to 376 barrer after UV treatment, while the CO_2_/CH_4_ ideal selectivity showed only a slight drop, from 39 to 35. These results were reproducibly observed in different samples as well (Table S11, Supporting Information). This behavior is remarkable given that crosslinked polymers generally exhibit a significant loss in permeability compared to precursor films, which is due to the decrease in their free volume from densification.^[^ ^5^, ^64^ ^]^ 6FDA‐DAM:DAA‐X membranes showcase up to 2 orders of magnitude higher gas permeabilities than commercial polymer membranes such as Matrimid, CA, and PSf, while still exhibiting selectivity values that are comparable. Of note, 6FDA‐DAM that was UV‐irradiated for 12 h (to serve as a control experiment) exhibited gas transport properties that were almost identical to those of its precursor. This control experiment highlights that the improved gas separation performance of 6FDA‐DAM:DAA‐X is mainly attributed to the formation of dianthracene units through UV irradiation.

A noticeable relationship between the permeability ratio of UV‐irradiated to precursor membranes (i.e., P_UV_/P_before_) and the effective diameter of gas molecules was not observed (Figure 4d). This behavior is in sharp contrast to the previous reports showing a positive correlation between the permeability ratio and the size of gas molecules when a membrane exhibited an enhanced FFV by incorporating additives such as oligomers or nanoparticles.^[^ ^65^, ^66^ ^]^ Since enhancing FFV will typically lead to a larger increase in permeability for larger gases compared to smaller gases, P_UV_/P_before_ will typically also be larger for larger gases. This usual trend can lead to lower diffusivity selectivity. However, the lack of a clear trend between P_UV_/P_before_ and the effective diameter of gas molecules for UV‐irradiated PIM‐PI films suggests a unique role from the IFV of dianthracene linkages (i.e., IFV size of 3.6 to 4.7 Å), which will be considered next.

To further gain insight into the gas transport properties, sorption and diffusion coefficients of 6FDA‐DAM:DAA and 6FDA‐DAM:DAA‐48 were acquired based on the sorption–diffusion model.^[^ ^3^ ^]^ 6FDA‐DAM:DAA‐48 exhibited a higher sorption capacity than 6FDA‐DAM:DAA for both CO_2_ and CH_4_ at all pressure ranges (Figure 4e), which can be explained by the enhanced microporosity in 6FDA‐DAM:DAA‐48 that increases the sorption capacity. The dual‐mode sorption model was used to fit each sorption isotherm and the model parameters were determined based on a precise nonlinear optimization method reported in previous studies (Table S12, Supporting Information).^[^ ^33^, ^34^, ^67^ ^]^ The product of the Langmuir affinity constant (*b*) and the Langmuir capacity constant (*C′_H_*) accounts for the contributions from excess free volume domains. This nonequilibrium contribution (*C′_H_b*) was higher for 6FDA‐DAM:DAA‐48 than 6FDA‐DAM:DAA, which is consistent with the specific surface area analyses discussed earlier.^[^ ^34^ ^]^ Furthermore, an increase in Henry's constant (*k_D_*) was observed for 6FDA‐DAM:DAA‐48 compared to 6FDA‐DAM:DAA, which indicates changes in the chemical composition of the polymer (i.e., dianthracene formation after UV exposure).^[^ ^34^ ^]^ For CO_2_, 6FDA‐DAM:DAA‐48 displayed a 78% and 67% increase in diffusion coefficient and sorption coefficient, respectively, from its precursor, resulting in a 224% increase in permeability coefficient (Figure 4f and Table S13, Supporting Information). CO_2_/CH_4_ diffusion selectivity ($D_{{\mathrm{CO}}_{2}}$/$D_{{\mathrm{CH}}_{4}}$) was enhanced for 6FDA‐DAM:DAA‐48 by 35% compared to the 6FDA‐DAM:DAA precursor while CO_2_/CH_4_ sorption selectivity ($S_{{\mathrm{CO}}_{2}}$/$S_{{\mathrm{CH}}_{4}}$) was reduced by 33%. The increase in diffusion selectivity is attributed to the size‐sieving nature of the resulting dianthracene linkage, while the decrease in sorption selectivity can be explained from the loss of π–π interactions between the anthracene unit and CO_2_ after UV‐induced branching.^[^ ^68^ ^]^ The overall change in both sorption and diffusion behavior in 6FDA‐DAM:DAA‐48 is responsible for its enhanced CO_2_ permeability and minimal loss in CO_2_/CH_4_ selectivity compared to the 6FDA‐DAM:DAA precursor.

**Figure 5:**
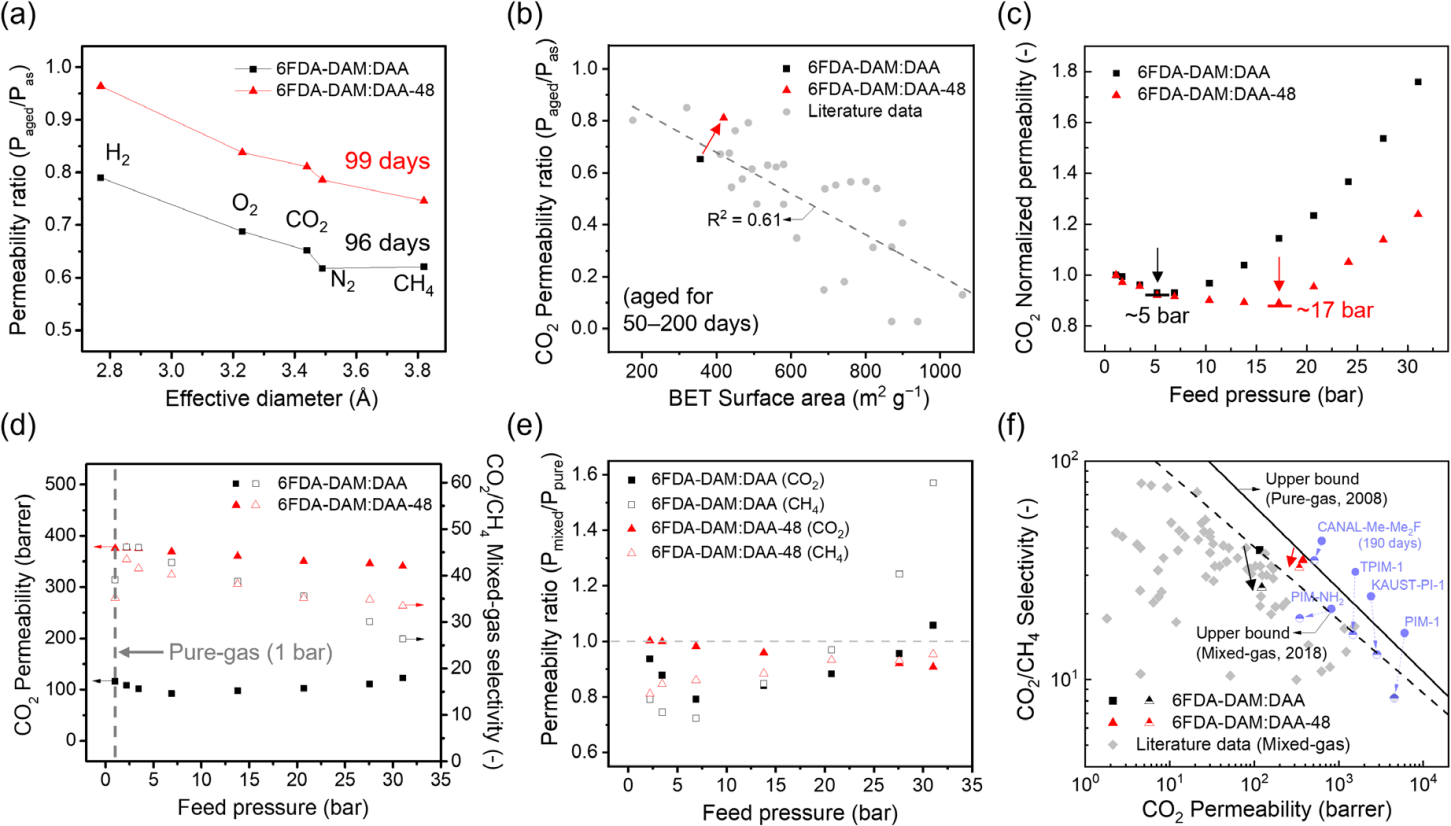
Physical aging and plasticization data for 6FDA‐DAM:DAA‐48 membranes (DAM:DAA = 5:5). a) Permeability ratio of aged 6FDA‐DAM:DAA (for 96 days) and 6FDA‐DAM:DAA‐48 membranes (for 99 days) (P_aged_) to as‐prepared ones (P_as_) versus effective diameter of gas molecules and b) the corresponding CO_2_ permeability ratio of aged samples to as‐prepared ones (P_aged_/P_as_) against BET surface area (from N_2_ sorption isotherms) plotted with literature data (gray circles) of 60–200 days aged PIM‐based membranes. The dotted gray line is a linear fit of the aging data of PIMs reported in the literature. c) Pure‐gas CO_2_ plasticization isotherms at 35 °C for 6FDA‐DAM:DAA and 6FDA‐DAM:DAA‐48 membranes plotted as normalized permeability (to that at 1 bar) as a function of feed pressure. The arrows indicate the plasticization pressure for each sample. d) CO_2_/CH_4_ mixed‐gas separation performance of 6FDA‐DAM:DAA and 6FDA‐DAM:DAA‐48 membrane films. Filled symbols represent CO_2_ permeability, while unfilled symbols represent CO_2_/CH_4_ mixed‐gas selectivity. e) Mixed‐ to pure‐gas permeability ratio (P_mixed_/P_pure_) for different total feed pressures from 2 to 31 bar, where the feed gas composition is CO_2_:CH_4_ = 50:50 mol% at 35 °C. Filled symbols represent P_mixed_/P_pure_ for CO_2_, while unfilled symbols represent P_mixed_/P_pure_ for CH_4_. f) CO_2_/CH_4_ mixed‐gas separation performance of 6FDA‐DAM:DAA and 6FDA‐DAM:DAA‐48 membrane films compared to literature data. Filled symbols indicate the pure‐gas separation performance at 1 bar and 35 °C, and half‐filled symbols indicate the mixed‐gas separation performance at 31 bar and 35 °C using a CO_2_:CH_4_ = 50:50 (mol%) feed gas. The pure gas upper bound is from ref. [15] while the mixed‐gas upper bound is from ref. [25]. Gray diamonds indicate the reported CO_2_/CH_4_ separation performances typically measured at 20 bar and 35 °C using a CO_2_:CH_4_ = 50:50 mol% feed gas.^[^ ^25^ ^]^ Blue circles indicate pure‐gas (2 bar, filled symbols) and mixed‐gas (CO_2_:CH_4_ = 50:50 mol%, 20 bar, half‐filled symbols) separation performance of several state‐of‐the‐art PIMs measured at 35 °C.

The long‐term stability of 6FDA‐DAM:DAA‐based membranes was also evaluated. PIMs are notorious for their poor long‐term stability due to physical aging, which results in a shrinkage of free volume elements and a concomitant decrease in gas permeability as time elapses.^[^ ^26^, ^29^, ^69^ ^]^ For example, a bulk sample of PIM‐1, which is the archetypal PIM, exhibits a 25% decrease in CO_2_ permeability after 53 days of aging.^[^ ^70^ ^]^ After ∼100 days of aging, both 6FDA‐DAM:DAA and 6FDA‐DAM:DAA‐48 exhibited a more pronounced decrease in gas permeability for larger gas molecules due to the reduction of larger free volume elements in the free volume size distribution (**Figure** **5**a),^[^ ^30^ ^]^ which is consistent with previous studies.^[^ ^71^ ^]^ Compared to the as‐prepared 6FDA‐DAM:DAA‐48 membrane film, the aged membrane film displayed a 23% boost in H_2_/CH_4_ selectivity, while the loss in H_2_ permeability was only 3%. H_2_/CH_4_ possesses the largest size difference among the investigated gas pairs and thus would exhibit the largest change in selectivity with aging. The aged 6FDA‐DAM:DAA‐48 membrane exhibited excellent H_2_/CH_4_ separation performance, surpassing the 2008 Robeson upper bound (Figure S23 and Table S14, Supporting Information).^[^ ^15^ ^]^

In general, physical aging is more accelerated for glassy polymers with a higher microporosity (or FFV) based on the Struik model.^[^ ^72^ ^]^ This behavior is evidenced by the literature data (Figure 5b; Table S15, Supporting Information), which reveals that PIMs with higher BET surface areas (calculated from N_2_ sorption isotherms) generally experience more intensified aging by showing a lower CO_2_ permeability ratio of the aged sample (for 60–200 days) to the as‐prepared one (P_aged_/P_as_). Interestingly, however, 6FDA‐DAM:DAA‐48 exhibited a less pronounced reduction in gas permeability after aging compared to the control 6FDA‐DAM:DAA despite its higher microporosity. This unusual behavior is ascribed to the ladder‐branched structure of 6FDA‐DAM:DAA‐48 that constrains the chain relaxation and free volume shrinkage.^[^ ^40^, ^41^, ^73^, ^74^ ^]^ It should be noted that although 6FDA‐DAM:DAA‐48 appears to exhibit superior aging resistance compared to literature data, the aging trend can vary substantially depending on film thickness, aging time, and testing conditions.^[^ ^30^, ^64^ ^]^

In addition to physical aging, plasticization has prevented the industrial deployment of developed polymer membrane materials due to reduced diffusion selectivity in mixture separations containing strongly sorbing gases (e.g., CO_2_/CH_4_).^[^ ^28^ ^]^ A common technique for evaluating plasticization in polymer membranes is the high‐pressure pure‐gas permeation test.^[^ ^34^ ^]^ Type III permeability isotherms were observed for both 6FDA‐DAM:DAA and 6FDA‐DAM:DAA‐48, while the pressure onset at which permeability began to increase with increasing feed pressure (i.e., the plasticization pressure) was significantly shifted to a higher pressure (≈17 bar) for 6FDA‐DAM:DAA‐48 compared to ≈5 bar for 6FDA‐DAM:DAA (Figure 5c).^[^ ^28^ ^]^ This enhanced plasticization resistance for 6FDA‐DAM:DAA‐48, which can be gleaned from the higher plasticization pressures reported for this polymer compared to other PIM‐PIs (e.g., KAUST‐PI‐1: 2 bar, KAUST‐PI‐5: 10 bar, PIM‐6FDA‐OH: 7 bar, and 6FDA‐DAM: 16 bar),^[^ ^28^ ^]^ is attributed to the ladder‐branching of 6FDA‐DAM:DAA that likely reduces cooperative chain motions.^[^ ^26^ ^]^ This observation was further supported by pressure‐dependent equimolar CO_2_/CH_4_ mixture permeation tests (total feed pressures from 2 to 31 bar, CO_2_:CH_4_ = 50:50 mol% at 35 °C; Figure 5d). Both 6FDA‐DAM:DAA and 6FDA‐DAM:DAA‐48 displayed an improved CO_2_/CH_4_ mixed‐gas selectivity at 2 bar compared to the pure‐gas case due to the competitive sorption effect, in which the presence of the strongly‐sorbing CO_2_ hinders the sorption of CH_4_ (Table S16, Supporting Information).^[^ ^24^, ^75^ ^]^ However, 6FDA‐DAM:DAA showed a significant reduction in mixed‐gas selectivity above 5 bar, while the selectivity decline was less pronounced for 6FDA‐DAM:DAA‐48.

To decouple the effects of competitive sorption and plasticization, the ratio of permeability evaluated under mixed‐gas and pure‐gas conditions (P_mixed_/P_pure_) was plotted as a function of the feed pressure (Figure 5e). For both membrane films, mixed‐gas CO_2_ permeability decreased with increasing feed pressure due to a decrease in the CO_2_ sorption coefficient with increasing pressure, which relates to dual‐mode effects.^[^ ^76^ ^]^ However, after a feed pressure of 5 bar, the mixed‐gas CH_4_ permeability of 6FDA‐DAM:DAA sharply increased and eventually exceeded the pure‐gas CH_4_ permeability by ≈60% at the highest pressure tested. In contrast, for 6FDA‐DAM:DAA‐48, both mixed‐gas CO_2_ and CH_4_ permeabilities were consistently less than pure‐gas permeabilities for all pressures tested (Table S16, Supporting Information). Thus, we conclude that only competitive sorption effects are pronounced in 6FDA‐DAM:DAA‐48 for the pressure range considered for mixed‐gas permeation, and plasticization effects are minimal.

Ultimately, the mixed‐gas CO_2_/CH_4_ separation performance of 6FDA‐DAM:DAA‐48 outperforms most polymers reported in literature including PIM‐1, PIM‐NH_2_, TPIM‐1, and KAUST‐PI‐1 (Table S17, Supporting Information). This performance surpasses the 2018 mixed‐gas upper bound even at the highest feed pressure for the mixture permeation test (31 bar) (Figure 5f).^[^ ^25^ ^]^ In addition, this performance is comparable with that of the state‐of‐the‐art CANAL‐Me‐Me_2_F, a polymer that requires multiple synthesis steps as well as long periods of time to exploit aging‐induced selectivity enhancements.^[^ ^24^ ^]^ These results clearly demonstrate the promising potential of the ladder‐branching strategy to improve the stability of microporous polymers for efficient gas separation under industrially relevant conditions.

Recent advances in photochemical process intensification and continuous‐flow platforms have demonstrated effective strategies to enhance photon utilization and mass/heat transfer.^[^ ^77^, ^78^, ^79^ ^]^ These developments may provide a practical pathway to significantly shorten irradiation times and improve scalability, thereby increasing the industrial relevance of the present ladder‐branched PIM‐PI modification approach. In addition, the excellent solution processability of these polymers facilitates their downscaling into thin, selective layers, such as submicron films or hollow fibers.^[^ ^4^, ^80^ ^]^ This versatility further supports the potential of the developed materials to be translated into scalable membrane fabrication routes for industrial gas separation.

## 3 Conclusion

We have demonstrated an efficient post‐synthetic modification to develop PIM‐PIs with enhanced gas separation performance and operational stability while maintaining solution processability. The key design strategy is incorporating a UV‐reactive monomer into the PIM‐PI to form a ladder‐branched structure. 6FDA‐DAM:DAA containing the anthracene unit was synthesized as a precursor PIM‐PI, and after UV exposure, the PIM‐PI showed a ladder‐branched structure that maintained solution processability. The resultant pentiptycene‐like dianthracene linkages increased both the microporosity and ultramicroporosity of the PIM‐PIs. As a result, the PIM‐PIs after UV exposure displayed significantly enhanced gas permeability with a marginal loss in selectivity, which has been rarely observed in common crosslinking modification of PIMs. Sorption–diffusion analyses confirmed that the IFV of the dianthracene linkage is responsible for the enhanced diffusion selectivity and gas permeability of the modified membranes. More importantly, the branched structure mitigated physical aging and penetrant‐induced plasticization in the PIM‐PIs, resulting in a high‐performance membrane for CO_2_/CH_4_ mixture separation surpassing the 2018 mixed‐gas upper bound. This strategy is expected to be applicable to other anthracene‐containing polyimides, thereby providing opportunities to further expand the scope of PIM‐PI chemistry. Future work will focus on converting ladder‐branched PIM‐PIs into industrially relevant thin‐film forms, including thin‐film composite and hollow‐fiber membranes. Beyond gas separation, we believe that this design strategy would be effective in fine‐tuning the microporosity, stability, and solubility of PIMs for additional molecular separation applications that require these features, such as organic solvent nanofiltration/reverse osmosis, aqueous separations, and flow batteries.

## 4 Experimental Section

### Materials

4,4′‐(Hexafluoroisopropylidene) diphthalic anhydride (6FDA, 99.5%) was purchased from Daken Chemical (China). Acetic anhydride (99.5%), β‐picoline (99%), methanol (MeOH, 99.8%), chloroform (CHCl_3_, 99.5%), *N*‐methyl‐2‐pyrrolidone (NMP, 99.5%, anhydrous), tetrahydrofuran (THF, 99.9%), and 2,4‐diaminomesitylene (DAM, 96%) were purchased from Sigma Aldrich (USA). 2,6‐Diaminoanthracene (DAA, 97%) was purchased from Ambeed (USA). DAM was purified by vacuum sublimation at 80 °C overnight with a cold trap consisting of dry ice, and all other chemicals were used as received. All gases with ultrahigh purity (>99.99%) were purchased from Linde US (USA).

### Synthesis of 6FDA‐DAM:DAA Copolyimides

6FDA‐DAM:DAA copolyimides (DAM:DAA molar ratio = 1:0, 9:1, 7:3, and 5:5) were synthesized by forming a poly(amic acid) followed by chemical imidization. Synthesis parameters are summarized in Table S1 (Supporting Information). Briefly, a certain amount of DAM and DAA monomers was dissolved in a round‐bottom flask containing NMP under N_2_ purge at room temperature. After dissolving the diamine monomers, the reaction flask was submerged in an ice bath. After 30 min, 6FDA monomer was added to the reactor to generate the poly(amic acid). During this reaction, the ice bath was removed after ≈3 h to raise the solution temperature to room temperature. After 24 h, β‐picoline and acetic anhydride were added to the reactor to convert the poly(amic acid) precursor to a polyimide. The mixture was stirred for another 24 h at room temperature. Finally, the resultant polyimide was precipitated in methanol, washed with methanol three times by filtration, and then dried in a vacuum oven at 120 °C for 24 h (yield: 80–90%).

### UV Irradiation to 6FDA‐DAM:DAA Copolyimides

To convert anthracene into dianthracene by [4+4] cycloaddition, a vial containing 20 mL of 6FDA‐DAM:DAA solution (DAM:DAA = 1:0, 9:1, 7:3, and 5:5, 1 w/v%, solvent: CHCl_3_) was exposed to UV light (365 nm) generated by a UV flashlight (36 W, SV43, Alonefire, China). The distance between the vial and the flashlight was ∼1.5 cm. After a certain irradiation time (X = 12, 24, 36, and 48 h), the resultant 6FDA‐DAM:DAA‐X solution was acquired and used to cast a dense membrane film. Powder samples were prepared by precipitating the UV‐treated solution in methanol, washing with methanol three times by filtration, and then drying in a vacuum oven at 120 °C for 24 h (yield: 70–80%). A schematic illustration and a photo image of UV irradiation of 6FDA‐DAM:DAA solutions are shown in Figure S9 (Supporting Information).

### Preparation of Dense Membranes

To prepare dense membranes, a certain amount of 6FDA‐DAM:DAA powder (DAM:DAA = 1:0, 9:1, 7:3, and 5:5) was dissolved in CHCl_3_ to prepare a 4 w/v% solution. The prepared solution (5 mL) was cast into a glass petri dish (5 cm diameter) and covered with a glass cover. After 2 days of solvent evaporation in a fume hood, a freestanding 6FDA‐DAM:DAA film (thickness = 60 ± 10 µm) was collected, immersed in methanol for 24 h, and then dried in a vacuum oven at 120 °C for 24 h before use. For the UV‐treated 6FDA‐DAM:DAA (i.e., 6FDA‐DAM:DAA‐X), the as‐prepared solution (15 mL) after UV irradiation was cast into a glass petri dish (5 cm diameter) and covered with a glass cover. After 4 days of solvent evaporation in a fume hood, a freestanding 6FDA‐DAM:DAA‐X film (thickness = 50 ± 10 µm) was collected, immersed in methanol for 24 h, and then dried in a vacuum oven at 120 °C for 24 h before use. For the aging tests, films were stored at room temperature (≈23 °C) and relative humidity of 20–40% under dark and ambient conditions.

### Characterization

Fourier transform infrared (FT‐IR) spectra were obtained using an FT‐IR spectrometer (ALPHA, Bruker, USA). ^1^H nuclear magnetic resonance (NMR) spectra were recorded with a 400 MHz Avance III HD spectrometer (Bruker, USA). To analyze the weight loss of polymer samples, a thermogravimetric analyzer (TGA, TGA550, TA Instruments, USA) was employed, with the temperature increasing under a nitrogen purge at a rate of 10 °C min^−1^ up to 800 °C. Mechanical properties of the polyimide films were evaluated using a universal testing machine (UTM; AGS‐J, Shimadzu, Japan). Dog‐bone‐shaped specimens were prepared in accordance with ASTM D638 Type V specifications. Each tensile test was carried out at a cross‐head speed of 5 mm min^−1^, and at least four specimens were tested for each sample. Microporosity of copolyimides was studied using N_2_ and CO_2_ sorption at 77 and 273 K, respectively, using polymer powders, respectively, on a physisorption analyzer (3Flex, Micromeritics, USA). Pore size distributions were determined using a non‐local density functional theory (NLDFT). Before sorption measurements, samples were degassed at 120 °C for 12 h. Brunauer–Emmett–Teller (BET) surface areas were determined from the N_2_ sorption isotherms, using a specific relative pressure range based on previously established criteria.^[^ ^52^ ^]^ Langmuir surface areas were calculated from the CO_2_ sorption isotherm within an absolute pressure range of 50–300 mmHg, according to the software's default settings (Flex Version 6.01).^[^ ^52^ ^]^ CO_2_ sorption isotherms were also collected at 298 K, and the isosteric heat of adsorption for CO_2_ was determined from the CO_2_ sorption isotherms at both 273 and 298 K using the same software. Molecular weights of copolyimides were obtained using a gel permeation chromatography (GPC) instrument (Agilent 1260 Infinity, USA) equipped with an LC‐5060 (LabACE, Japan) recycling preparative high‐performance liquid chromatography (HPLC) containing refractive index and ultraviolet detectors, and a JAIGEL‐2.5HR column from Japan Analytical Industry (Japan). The system was calibrated with polystyrene standards between 1.7 and 3150 kg mol^−1^. All runs were conducted in HPLC‐grade tetrahydrofuran at a 1.0 mL min^−1^ flow rate and 35 °C. Molecular weight values were calculated using the ChemStation GPC data analysis software (rev. B.01.01) based on the refractive index signal. A UV–vis spectrometer (Cary 60, Agilent Technologies, USA) was used to obtain UV–vis spectra of copolyimide solutions. Wide‐angle X‐ray scattering (WAXS) patterns were collected under a vacuum of 0.08 mbar using a SAXSLAB apparatus (Xenocs, France) with a PILATUS3 R 300K detector (Dectris, USA) and a Rigaku 002 microfocus X‐ray source (Rigaku, Japan). Each pattern was acquired over 1200 s and was presented as intensity *I(q)* against scattering wavevector *q*, which was calculated as follows:

(1) $$q=\frac{4\pi \sin\theta }{\lambda }$$

where *θ* is Bragg's angle and *λ* is the wavelength of the X‐ray source. The WAXS peaks for each pattern were identified using the “Gaussian peak + slope background” function available in the SAXSGUI software from the MIT Materials Research Laboratory.

### Pure‐Gas Permeation

Samples for gas permeation tests were prepared by mounting pieces of the as‐prepared films onto circular brass supporting disks with a small central hole. The film edges were sealed with epoxy (Devcon 5 min Epoxy), leaving only the defined active area exposed for permeation measurements. The effective membrane area was determined to be 0.20–0.25 cm^2^ by analyzing the scanned images of the mounted membranes with ImageJ software, excluding the epoxy‐sealed portion. The pure‐gas permeabilities (H_2_, O_2_, CO_2_, N_2_, and CH_4_) of 6FDA‐DAM:DAA and 6FDA‐DAM:DAA‐X membranes were measured using an automated constant‐volume variable‐pressure permeation system (Maxwell Robotics, USA). To prevent any leakage between the upstream (feed) and downstream (permeate), all membrane samples were secured to brass supports with epoxy adhesive. Before permeation tests, the entire system was evacuated for 8 h. Each gas was injected into the feed side while the upstream side was purged with He, and the system was evacuated for 1 h after each gas was tested. All permeation tests were conducted at 35 °C for 1 h (for H_2_, O_2_, and CO_2_) or 2.5 h (for N_2_ and CH_4_) with an upstream pressure of ≈1 bar and a downstream pressure below 9.5 torr. The permeability coefficient (*P*, unit: barrer, 1 barrer = 10^−10^ cm^3^ (STP) cm cm^−2^ s^−1^ cmHg^−1^) was calculated as follows:

(2) $$P=\frac{Vl}{\Delta pRTA}\left[{\left(\frac{dp}{dt}\right)}_{ss}-{\left(\frac{dp}{dt}\right)}_{leak}\right]$$

where *V* (cm^3^) is the volume of the permeate side, *R* is the gas constant, *l* (cm) is the membrane thickness, *∆p* (cmHg) is the pressure difference between the feed and permeate side, *A* (cm^2^) is the membrane area, *T* (K) is the temperature, (*dp⁄dt*)*_ss_* is the rate of pressure increase at steady‐state, and (*dp⁄dt*)*_leak_* is the leak rate measured before the permeation tests for 0.5 h.

The ideal selectivity (*α_A/B_*) was calculated as the ratio of the permeability coefficient of the two single‐component gases (A and B) as follows:

(3) $$\alpha_{A/B}=\frac{P_{A}}{P_{B}}$$

### Mixed‐Gas Permeation

Mixed‐gas permeation tests for CO_2_/CH_4_ mixtures were conducted at 35 °C using an automated constant‐volume variable‐pressure permeation system (Maxwell Robotics, USA) connected to a gas chromatograph (GC, Agilent 7890B, Agilent Technologies, USA). The feed gas was prepared by mixing each gas component with a mass flow controller (MFC) prior to the tests. Similar to the pure‐gas permeation tests, the downstream side of the permeation cell was evacuated for 8 h before each experiment. The tests were carried out by injecting the feed gas. Once the pressure increase rate reached a steady state (typically 2–3 h), the permeate was collected and its composition analyzed using the GC. The mixed‐gas CO_2_/CH_4_ selectivity was then calculated as follows:

(4) $$\alpha_{mix}=\frac{y_{CO_{2}}/y_{CH_{4}}}{x_{CO_{2}}/x_{CH_{4}}}$$

where y and x are the mole fractions of each gas in the permeate and feed, respectively. The upstream flow rate was adjusted between 150 and 250 sccm depending on the feed pressure.

### High‐Pressure Pure‐Gas Sorption

High‐pressure sorption isotherms for CO_2_ and CH_4_ were obtained using an automated dual‐volume, dual‐transducer pressure decay sorption system (Maxwell Robotics, USA). Approximately 0.10–0.15 g of polymer sample was placed in a sealed metal cell, and the system was evacuated for 10 h to degas the samples. Sorption analyses were conducted at 35 °C up to ≈50 bar. The amount of gas sorbed by the sample at each pressure step was calculated based on the pressure change from initial to equilibrium conditions in the sample chamber. The dual‐mode sorption model was applied to fit the resulting sorption isotherms as follows:
(5)

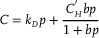

where *C* is the concentration of penetrant in the polymer (cm^3^ (STP) cm^−3^ (polymer)), *k_D_* is Henry's constant (cm^3^ (STP) cm^−3^ (polymer) atm^−1^), *p* is the equilibrium pressure (atm), *C′_H_* is the Langmuir capacity constant (cm^3^ (STP) cm^−3^ (polymer)), and *b* is the Langmuir affinity constant (atm^−1^).

Sorption coefficients at 1 bar (*p* = 1 bar) were obtained by sorption isotherms fitted to the dual‐mode sorption model as follows:

(6)

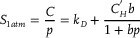




## Conflict of Interest

The authors declare no conflict of interest.

## Supporting information



Supporting Information

## Data Availability

The data that support the findings of this study are available on request from the corresponding author. The data are not publicly available due to privacy or ethical restrictions.
