# Supplementary material for: Solution‐Processable, Ladder‐Branched Polyimides of Intrinsic Microporosity by [4+4] Cycloaddition for Membrane Gas Separation
Source: Adv Mater. 2025 Oct 15;38(4):e13892. doi: 10.1002/adma.202513892 (PMC12810612; doi:10.1002/adma.202513892)
Supplement: Supplementary file 1 — Supporting Information [file ADMA-38-e13892-s001.docx]

Supporting Information

**Solution-Processable, Ladder-Branched Polyimides of Intrinsic Microporosity by [4+4] Cycloaddition for Membrane Gas Separation**

Tae Hoon Lee, Pablo A. Dean, Jing Ying Yeo, and Zachary P. Smith*

Prof. T. H. Lee, Dr. P. A. Dean, Dr. J. Y. Yeo, Prof. Z. P. Smith

Department of Chemical Engineering, Massachusetts Institute of Technology, Cambridge, Massachusetts 02139, United States
E-mail: zpsmith@mit.edu (Z. P. Smith)

Prof. T. H. Lee
Department of Future Energy Engineering, Sungkyunkwan University, Suwon 16419, Republic of Korea

**Figure S1.** ^1^H NMR spectra of 6FDA-DAM:DAA copolyimides with different DAM:DAA ratios.

**Figure S2.** FT-IR spectra of 6FDA-DAM:DAA copolyimides with different DAM:DAA ratios.

**Figure S3.** TGA curves of 6FDA-DAM:DAA copolyimides with different DAM:DAA ratios.

**Figure S4.** GPC curves of 6FDA-DAM:DAA copolyimides with different DAM:DAA ratios.

**Figure S5.** N_2_ adsorption–desorption isotherms of 6FDA-DAM:DAA copolyimides with different DAM:DAA ratios measured at 77 K. Filled and unfilled symbols represent adsorption and desorption, respectively.

**Figure S6.** CO_2_ adsorption–desorption isotherms of 6FDA-DAM:DAA copolyimides with different DAM:DAA ratios measured at (a) 273 K and (b) 298 K. Filled and unfilled symbols represent adsorption and desorption, respectively.

**Figure S7.** Isosteric heats of adsorption for 6FDA-DAM:DAA copolyimides with different DAM:DAA ratios obtained from CO_2_ sorption isotherms at 273 and 298 K (*cf.* **Figure S6**).^[1, 2]^

**Figure S8.** UV-vis absorbance spectra of 6FDA-DAM:DAA copolyimides with different DAM:DAA ratios (solvent: CHCl_3_, 0.01 w/v%).

**Figure S9.** (a) Schematic illustration and (b) picture of UV (365 nm) irradiation of a 6FDA-DAM:DAA solution (DAM:DAA = 5:5, solvent: CHCl_3_ and 1 w/v%). After taking the picture, the system was fully covered using aluminum foil to prevent exposure to any external light sources.

**Figure S10.** Photo images of UV-treated 6FDA-DAM:DAA solution (DAM:DAA = 5:5) with different irradiation times (solvent: CHCl_3_, 1 w/v%).

**Figure S11.** TGA curves of 6FDA-DAM:DAA and 6FDA-DAM:DAA-48 copolyimides (DAM:DAA = 5:5).

**Figure S12.** Representative stress–strain curves of 6FDA-DAM:DAA and 6FDA-DAM:DAA-48 copolyimides (DAM:DAA = 5:5) and the corresponding mechanical properties.

**Figure S13.** CO_2_ adsorption–desorption isotherms of 6FDA-DAM:DAA and 6FDA-DAM:DAA-48 copolyimides meausred at 298 K (DAM:DAA = 5:5). Filled and unfilled symbols represent adsorption and desorption, respectively.

**Figure S14.** Isosteric heats of adsorption for 6FDA-DAM:DAA and 6FDA-DAM:DAA-48 copolyimides obtained from CO_2_ sorption isotherms at 273 and 298 K (*cf.* **Figure S13** and **Figure 3b**, respectively).

**Figure S15.** UV-vis absorbance spectra of UV-treated 6FDA-DAM:DAA copolyimides with different DAM:DAA ratios of (a) 1:0, (b) 9:1, and (c) 7:3 (solvent: CHCl_3_, 0.01 w/v%).

**Figure S16.** ^1^H NMR spectra of UV-treated 6FDA-DAM:DAA copolyimides with different DAM:DAA ratios.

**Figure S17.** GPC curves of UV-treated 6FDA-DAM:DAA copolyimides with different DAM:DAA ratios of (a) 1:0, (b) 9:1, and (c) 7:3.

**Figure S18.** TGA curves of UV-treated 6FDA-DAM:DAA copolyimides with different DAM:DAA ratios.

**Figure S19.** N_2_ adsorption–desorption isotherms of UV-treated 6FDA-DAM:DAA copolyimides with different DAM:DAA ratios of (a) 1:0, (b) 9:1, and (c) 7:3 measured at 77 K. Filled and unfilled symbols represent adsorption and desorption, respectively.

**Figure S20.** CO_2_ adsorption–desorption isotherms of UV-treated 6FDA-DAM:DAA copolyimides with different DAM:DAA ratios of (a) 1:0, (b) 9:1, and (c) 7:3 measured at 273 K. Filled and unfilled symbols represent adsorption and desorption, respectively.

**Figure S21.** CO_2_ adsorption–desorption isotherms of UV-treated 6FDA-DAM:DAA copolyimides with different DAM:DAA ratios of (a) 1:0, (b) 9:1, and (c) 7:3 measured at 298 K. Filled and unfilled symbols represent adsorption and desorption, respectively.

**Figure S22.** Isosteric heats of adsorption for 6FDA-DAM:DAA copolyimides with different DAM:DAA ratios obtained from CO_2_ sorption isotherms at 273 and 298 K (*cf.* **Figure S20** and **Figure S21**, respectively).

**Figure S23.** Pure-gas separation performance of as-prepared and aged 6FDA-DAM:DAA (for 96 days) and 6FDA-DAM:DAA-48 (for 99 days) membranes (DAM:DAA = 5:5) plotted on upper bound plots for (a) H_2_/CH_4_, (b) O_2_/N_2_, and (c) CO_2_/CH_4_ measured at 35 °C and 1 bar. Gray triangles represent commercial polymer membranes.

**Table S1.** Synthesis parameters of 6FDA-DAM:DAA polyimides with different DAM:DAA ratios.

| DAM:DAA ratio | 6FDA | DAM | DAA | NMP | β-Picoline | Acetic anhydride |
| --- | --- | --- | --- | --- | --- | --- |
| 1:0 | 9.00 g  (20 mmol) | 3.04 g  (20 mmol) | - | 50 ml | 2 g | 20 g |
| 9:1 | 9.00 g  (20 mmol) | 2.70 g  (18 mmol) | 0.417 g  (2 mmol) | 50 ml | 2 g | 20 g |
| 7:3 | 9.00 g  (20 mmol) | 2.10 g  (14 mmol) | 1.25 g  (6 mmol) | 50 ml | 2 g | 20 g |
| 5:5 | 9.00 g  (20 mmol) | 1.50 g  (10 mmol) | 2.08 g  (10 mmol) | 60 ml | 2 g | 20 g |

**Table S2.** GPC-based molecular weight of 6FDA-DAM:DAA copolyimides with different DAM:DAA ratios.

| DAM:DAA ratio | M_n_ (g mol^−1^) | M_w_ (g mol^−1^) | PDI (-) |
| --- | --- | --- | --- |
| 1:0 | 99,000 | 197,000 | 2.0 |
| 9:1 | 44,000 | 84,000 | 1.9 |
| 7:3 | 23,000 | 41,000 | 1.8 |
| 5:5 | 27,000 | 75,000 | 2.8 |

**Table S3.** Specific surface areas of 6FDA-DAM:DAA copolyimides with different DAM:DAA ratios. BET surface area (SA_N2_) was calculated from N_2_ adsorption isotherms at 77 K while Langmuir surface area (SA_CO2_) was calculated from CO_2_ adsorption isotherms at 273 K.

| DAM:DAA ratio | SA_N2_ (cm^2^ g^−1^) | SA_CO2_ (cm^2^ g^−1^) |
| --- | --- | --- |
| 1:0 | 489 | 124 |
| 9:1 | 439 | 126 |
| 7:3 | 415 | 113 |
| 5:5 | 356 | 115 |

**Table S4.** Conversion of anthracene into dianthracene in UV-treated 6FDA-DAM:DAA (DAM:DAA = 5:5) with different irradiation times, calculated from UV-vis and NMR spectra.

| Irradiation time (h) | Conversion (%) | |
| --- | --- | --- |
|  | UV-vis | NMR |
| 0 | 0 | 0 |
| 12 | 8 | 5 |
| 24 | 29 | 34 |
| 36 | 45 | 50 |
| 48 | 64 | 63 |

**Table S5.** GPC-based molecular weights of UV-treated 6FDA-DAM:DAA (DAM:DAA = 5:5) with different irradiation times.

| Irradiation time (h) | M_n_ (g mol^−1^) | M_w_ (g mol^−1^) | PDI (-) |
| --- | --- | --- | --- |
| 0 | 27,000 | 75,000 | 2.8 |
| 12 | 26,000 | 95,000 | 3.7 |
| 24 | 29,000 | 172,000 | 5.9 |
| 36 | 26,000 | 287,000 | 11.0 |
| 48 | 32,000 | 357,000 | 11.2 |

**Table S6**. Specific surface areas of 6FDA-DAM:DAA and 6FDA-DAM:DAA-48 copolyimides (DAM:DAA = 5:5). BET surface area (SA_N2_) was calculated from N_2_ adsorption isotherms at 77 K while Langmuir surface area (SA_CO2_) was calculated from CO_2_ adsorption isotherms at 273 K.

| Sample | SA_N2_ (cm^2^ g^−1^) | SA_CO2_ (cm^2^ g^−1^) |
| --- | --- | --- |
| 6FDA-DAM:DAA | 356 | 115 |
| 6FDA-DAM:DAA-48 | 453 | 151 |

**Table S7.** Conversion of anthracene into dianthracene in UV-treated 6FDA-DAM:DAA copolyimides with different DAM:DAA ratios, calculated from UV-vis and NMR spectra. The data for 6FDA-DAM:DAA (DAM:DAA = 5:5) are reproduced from Table S4 for comparison purposes.

| DAM:DAA ratio | Conversion (%) | |
| --- | --- | --- |
|  | UV-vis | NMR |
| 1:0-12h | N/A | N/A |
| 9:1-12h | 79 | 73 |
| 7:3-36h | 69 | 60 |
| 5:5-48h | 64 | 63 |

**Table S8.** GPC-based molecular weights of UV-treated 6FDA-DAM:DAA copolyimides with different DAM:DAA ratios. The data for 6FDA-DAM:DAA (DAM:DAA = 5:5) are reproduced from Table S5 for comparison purposes.

| DAM:DAA ratio | M_n_ (g mol^−1^) | M_w_ (g mol^−1^) | PDI (-) |
| --- | --- | --- | --- |
| Before UV | | |  |
| 1:0 | 99,000 | 197,000 | 2.0 |
| 9:1 | 44,000 | 84,000 | 1.9 |
| 7:3 | 23,000 | 41,000 | 1.8 |
| 5:5 | 27,000 | 75,000 | 2.8 |
| After UV | | |  |
| 1:0-12h | 42,000 | 106,000 | 2.5 |
| 9:1-12h | 48,000 | 246,000 | 5.5 |
| 7:3-36h | 22,000 | 486,000 | 21.7 |
| 5:5-48h | 32,000 | 357,000 | 11.2 |

**Table S9**. Specific surface areas of UV-treated 6FDA-DAM:DAA copolyimides with different DAM:DAA ratios. BET surface area (SA_N2_) was calculated from N_2_ adsorption isotherms at 77 K while Langmuir surface area (SA_CO2_) was calculated from CO_2_ adsorption isotherms at 273 K. The data for 6FDA-DAM:DAA (DAM:DAA = 5:5) and 5:5-48h are reproduced from **Table S6** for comparison purposes.

| DAM:DAA ratio | SA_N2_ (cm^2^ g^−1^) | SA_CO2_ (cm^2^ g^−1^) | |
| --- | --- | --- | --- |
| Before UV | | |  |
| 1:0 | 489 | 124 | |
| 9:1 | 439 | 126 | |
| 7:3 | 415 | 113 | |
| 5:5 | 356 | 115 | |
| After UV | | |  |
| 1:0-12h | 441 | 148 | |
| 9:1-12h | 458 | 140 | |
| 7:3-36h | 450 | 140 | |
| 5:5-48h | 453 | 151 | |

**Table S10.** Pure-gas separation performance of 6FDA-DAM:DAA and 6FDA-DAM:DAA-X membranes measured at 35 °C and 1 bar.

| Before UV | | | | |
| --- | --- | --- | --- | --- |
| DAM:DAA ratio | 1:0 | 9:1 | 7:3 | 5:5 |
| Gas | Gas permeability (barrer) | | | |
| H_2_ | 540 | 332 | 228 | 137 |
| O_2_ | 163 | 73 | 39 | 21 |
| CO_2_ | 1055 | 458 | 246 | 116 |
| N_2_ | 49 | 20 | 8.6 | 4.3 |
| CH_4_ | 56 | 18 | 7.3 | 3.0 |
| Gas pair | Ideal selectivity (-) | | | |
| H_2_/CH_4_ | 9.7 | 18 | 31 | 46 |
| O_2_/N_2_ | 3.3 | 3.7 | 4.5 | 4.9 |
| CO_2_/CH_4_ | 19 | 25 | 33 | 39 |
| After UV | | | | |
| DAM:DAA ratio | 1:0-12h | 9:1-12h | 7:3-36h | 5:5-48h |
| Gas | Gas permeability (barrer) | | | |
| H_2_ | 605 | 672 | 630 | 411 |
| O_2_ | 201 | 157 | 111 | 67 |
| CO_2_ | 901 | 802 | 624 | 376 |
| N_2_ | 65 | 43 | 27 | 15 |
| CH_4_ | 50 | 34 | 21 | 11 |
| Gas pair | Ideal selectivity (-) | | | |
| H_2_/CH_4_ | 12 | 20 | 30 | 39 |
| O_2_/N_2_ | 3.1 | 3.6 | 4.1 | 4.4 |
| CO_2_/CH_4_ | 18 | 24 | 30 | 35 |

**Table S11.** Reproducibility of pure-gas separation performance of 6FDA-DAM:DAA and 6FDA-DAM:DAA-48 (DAM:DAA ratio = 5:5) measured at 35 °C and 1 bar.

| 6FDA-DAM:DAA | | | | | |
| --- | --- | --- | --- | --- | --- |
| Sample number | #1 | #2 | #3 | #4 | Average & Standard deviation |
| Gas | Gas permeability (barrer) | | | | |
| H_2_ | 137 | 110 | 112 | 91 | 113 ± 19 |
| O_2_ | 21 | 15 | 16 | 15 | 16 ± 3 |
| CO_2_ | 116 | 104 | 89 | 109 | 105 ± 11 |
| N_2_ | 4.3 | 3.3 | 2.9 | 3.6 | 3.5 ± 0.6 |
| CH_4_ | 3.0 | 2.8 | 2.3 | 2.5 | 2.7 ± 0.3 |
| Gas pair | Ideal selectivity (-) | | | | |
| H_2_/CH_4_ | 46 | 39 | 49 | 36 | 43 ± 6 |
| O_2_/N_2_ | 4.9 | 4.5 | 5.5 | 4.2 | 4.8 ± 0.6 |
| CO_2_/CH_4_ | 39 | 37 | 38 | 44 | 40 ± 3 |
| 6FDA-DAM:DAA-48 | | | | | |
| Sample number | #1 | #2 | #3 | #4 | Average & Standard deviation |
| Gas | Gas permeability (barrer) | | | | |
| H_2_ | 411 | 430 | 462 | 397 | 425 ± 28 |
| O_2_ | 67 | 51 | 64 | 58 | 60 ± 7 |
| CO_2_ | 376 | 422 | 372 | 410 | 395 ± 25 |
| N_2_ | 15 | 11 | 12 | 14 | 13 ± 2 |
| CH_4_ | 11 | 12 | 11 | 9.5 | 11 ± 1 |
| Gas pair | Ideal selectivity (-) | | | | |
| H_2_/CH_4_ | 39 | 36 | 42 | 42 | 40 ± 3 |
| O_2_/N_2_ | 4.4 | 4.6 | 5.3 | 4.1 | 4.6 ± 0.5 |
| CO_2_/CH_4_ | 35 | 35 | 34 | 43 | 37 ± 4 |

**Table S12.** Dual-mode sorption model parameters of 6FDA-DAM:DAA and 6FDA-DAM:DAA-48 membranes (DAM:DAA = 5:5) calculated from CH_4_ and CO_2_ sorption isotherms at 35 °C (**Figure 4e**).

| Sample | Gas | *k_D_* (cm^3^_STP_ cm^−3^_pol_ atm^−1^) | *C′_H_* (cm^3^_STP_ cm^−3^_pol_) | *b* (atm^−1^) | *C′_H_ b* (cm^3^_STP_ cm^−3^_pol_ atm^−1^) | *S_1 atm_* (cm^3^_STP_ cm^−3^_pol_ atm^−1^) |
| --- | --- | --- | --- | --- | --- | --- |
| 6FDA-DAM:DAA | CH_4_ | 0.51 | 27.9 | 0.05 | 1.4 | 1.7 |
|  | CO_2_ | 3.27 | 25.0 | 0.87 | 21.8 | 13.5 |
| 6FDA-DAM:DAA-48 | CH_4_ | 1.08 | 16.6 | 0.16 | 2.7 | 4.2 |
|  | CO_2_ | 3.73 | 27.3 | 1.12 | 30.6 | 22.6 |

**Table S13.** Diffusion, sorption, and permeability coefficients and corresponding selectivities of 6FDA-DAM:DAA and 6FDA-DAM:DAA-48 membranes (DAM:DAA = 5:5) measured at 35 °C and 1 bar.

| Sample | Gas | D (×10^−7^ cm^2^ s^−1^) | S_1 atm_ (cm^3^_STP_ cm^−3^_pol_ atm^−1^) | P (barrer) | D_CO2_/D_CH4_ | S_CO2_/S_CH4_ | P_CO2_/P_CH4_ |
| --- | --- | --- | --- | --- | --- | --- | --- |
| 6FDA-DAM:DAA | CH_4_ | 0.13 | 1.7 | 3.0 | 4.8 8.1 39 | | |
|  | CO_2_ | 0.63 | 13.5 | 116 |  |  |  |
| 6FDA-DAM:DAA-48 | CH_4_ | 0.17 | 4.2 | 11 | 6.5 5.4 35 | | |
|  | CO_2_ | 1.12 | 22.6 | 376 |  |  |  |

**Table S14.** Pure-gas separation performance of aged 6FDA-DAM:DAA (96 days) and 6FDA-DAM:DAA-48 membranes (99 days) compared to as-prepared (0 days) membranes.

| Sample | 6FDA-DAM:DAA | | 6FDA-DAM:DAA-48 | |
| --- | --- | --- | --- | --- |
| Gas | Gas permeability (barrer) | | | |
|  | 0 days | 96 days | 0 days | 99 days |
| H_2_ | 137 | 108 | 411 | 396 |
| O_2_ | 21 | 14 | 67 | 56 |
| CO_2_ | 116 | 76 | 376 | 305 |
| N_2_ | 4.3 | 2.7 | 15 | 12 |
| CH_4_ | 3.0 | 1.9 | 11 | 8.2 |
| Gas pair | Ideal selectivity (-) | | | |
|  | 0 days | 96 days | 0 days | 99 days |
| H_2_/CH_4_ | 46 | 58 | 39 | 48 |
| O_2_/N_2_ | 4.9 | 5.4 | 4.4 | 4.8 |
| CO_2_/CH_4_ | 39 | 41 | 35 | 37 |

**Table S15.** CO_2_ permeability ratios of aged samples to as-prepared samples (P_aged_/P_as_) against Brunauer–Emmett–Teller (BET) surface areas (by N_2_ sorption isotherms) for literature data of 60–200 days aged membrane films based on polymers of intrinsic microporosity (PIMs).

| Sample | Age (days) | P_as_ (barrer) | P_aged_ (barrer) | P_aged_/P_as_ | S_BET_ (m^2^ g^−1^) | Ref. |
| --- | --- | --- | --- | --- | --- | --- |
| PIM-1 | 155 | 6400 | 2000 | 0.31 | 820 | ^[3]^ |
| PIM-HPB | 150 | 3800 | 2390 | 0.62 | 537 | ^[4]^ |
| PIM-CH_3_-HPB | 150 | 2620 | 1630 | 0.62 | 560 | ^[4]^ |
| PIM-Br-HPB | 150 | 2130 | 1430 | 0.67 | 410 | ^[4]^ |
| PIM-CN-HPB | 150 | 2390 | 1300 | 0.54 | 440 | ^[4]^ |
| PIM-Trip-TB | 100 | 9709 | 3951 | 0.41 | 899 | ^[5]^ |
| PIM-Btrip-TB | 166 | 13200 | 4150 | 0.31 | 870 | ^[6]^ |
| TB-Ad-Me | 180 | 1820 | 635 | 0.35 | 615 | ^[7]^ |
| PIM-MP-TB | 118 | 3500 | 633 | 0.18 | 743 | ^[8]^ |
| SBFDA-DMN | 200 | 4700 | 703 | 0.15 | 688 | ^[9]^ |
| EA-DMN | 180 | 6520 | 3603 | 0.55 | 720 | ^[10]^ |
| CTB1-DMN | 60 | 1661 | 795 | 0.48 | 580 | ^[11]^ |
| CTB2-DMN | 60 | 948 | 546 | 0.58 | 469 | ^[11]^ |
| PIM-PI-TB-1 | 180 | 662 | 361 | 0.55 | 440 | ^[12]^ |
| PIM-PI-TB-2 | 180 | 595 | 376 | 0.63 | 580 | ^[12]^ |
| 6FDA-DAT1 | 150 | 120 | 102 | 0.85 | 320 | ^[13]^ |
| 6FDA-DAT2 | 150 | 210 | 160 | 0.76 | 450 | ^[13]^ |
| CANAL-Me-Me_2_F | 150 | 3700 | 590 | 0.17 | 1190 | ^[14]^ |
| CANAL-Me-S5F | 150 | 4000 | 520 | 0.13 | 1060 | ^[14]^ |
| CANAL-Me-S6F | 150 | 2900 | 80 | 0.03 | 940 | ^[14]^ |
| CANAL-Me-DHP | 158 | 3400 | 94 | 0.03 | 870 | ^[14]^ |
| CANAL-Me-2,2'-SBF | 160 | 2230 | 1070 | 0.48 | 508 | ^[15]^ |
| CANAL-Me-2,7-SBF | 170 | 2600 | 1400 | 0.54 | 691 | ^[15]^ |
| PIM-DBMP0.1 | 140 | 18900 | 10700 | 0.57 | 801 | ^[16]^ |
| PIM-DBMP0.25 | 127 | 21600 | 12200 | 0.56 | 760 | ^[16]^ |
| PIM-DBMP0.5 | 125 | 22200 | 11980 | 0.54 | 830 | ^[16]^ |
| PIM-SBI-IM3 | 121 | 1393 | 941 | 0.68 | 434 | ^[17]^ |
| PIM-SBI-IM5 | 92 | 2165 | 1330 | 0.61 | 495 | ^[17]^ |
| 6FDA-DAM:DAA | 96 | 116 | 76 | 0.66 | 356 | This study |
| 6FDA-DAM:DAA-48 | 99 | 376 | 305 | 0.81 | 453 |  |

**Table S16.** Pure-gas (1 bar) and mixed-gas (CO_2_:CH_4_ = 50:50 (mol%), 2–27 bar) separation performance of 6FDA-DAM:DAA and 6FDA-DAM:DAA-48 at 35 °C. Mixed-gas pressures are total pressure.

| Pressure (bar) | 6FDA-DAM:DAA | | 6FDA-DAM:DAA-48 | |
| --- | --- | --- | --- | --- |
|  | CO_2_ Permeability (barrer) | CO_2_/CH_4_ Selectivity (-) | CO_2_ Permeability (barrer) | CO_2_/CH_4_ Selectivity (-) |
| Pure-gas | | | | |
| 1 | 116 | 39 | 376 | 35 |
| Mixed-gas | | | | |
| 2 | 109 | 46 | 377 | 43 |
| 3 | 102 | 46 | 376 | 42 |
| 7 | 92 | 43 | 369 | 40 |
| 14 | 98 | 39 | 361 | 38 |
| 20 | 103 | 36 | 351 | 35 |
| 27 | 111 | 30 | 346 | 35 |
| 31 | 123 | 26 | 341 | 33 |

**Table S17.** Pure-gas (1 or 2 bar) and mixed-gas (CO_2_:CH_4_ = 50:50 (mol%), 20 or 27 bar) separation performance of several state-of-the-art PIMs measured at 35 °C. Mixed-gas pressures are total pressure.

| Sample | Condition | Pressure | P_CO2_ (barrer) | CO_2_/CH_4_ Selectivity | Ref. |
| --- | --- | --- | --- | --- | --- |
| CANAL-Me-Me_2_F | Pure-gas | 2 | 630 | 43 | ^[14]^ |
|  | Mixed-gas | 20 | 502 | 38 |  |
|  | Mixed-gas | 27 | 516 | 35 |  |
| PIM-1 | Pure-gas | 2 | 6034 | 16 | ^[18]^ |
|  | Mixed-gas | 20 | 4563 | 8.2 |  |
| KAUST-PI-1 | Pure-gas | 2 | 2420 | 24 | ^[18]^ |
|  | Mixed-gas | 20 | 2843 | 13 |  |
| TPIM-1 | Pure-gas | 2 | 1576 | 31 | ^[18]^ |
|  | Mixed-gas | 20 | 1475 | 16 |  |
| PIM-NH_2_ | Pure-gas | 2 | 819 | 21 | ^[19]^ |
|  | Mixed-gas | 20 | 346 | 19 |  |
| 6FDA-DAM:DAA | Pure-gas | 1 | 116 | 39 | This study |
|  | Mixed-gas | 20 | 103 | 36 |  |
|  | Mixed-gas | 27 | 111 | 30 |  |
| 6FDA-DAM:DAA-48 | Pure-gas | 1 | 376 | 35 | This study |
|  | Mixed-gas | 20 | 351 | 35 |  |
|  | Mixed-gas | 27 | 346 | 35 |  |

**References**

[1] T. H. Lee, B. K. Lee, S. Y. Yoo, H. Lee, W. N. Wu, Z. P. Smith, H. B. Park, *Nat. Commun.* **2023**, *14*, 8330.

[2] T. H. Lee, M. Balcik, W.-N. Wu, I. Pinnau, Z. P. Smith, *Sci. Adv.* **2024**, *10*, eadp6666.

[3] M. Alberto, R. Bhavsar, J. M. Luque-Alled, A. Vijayaraghavan, P. M. Budd, P. Gorgojo, *J. Membr. Sci.* **2018**, *563*, 513.

[4] M. Carta, P. Bernardo, G. Clarizia, J. C. Jansen, N. B. McKeown, *Macromolecules* **2014**, *47*, 8320.

[5] M. Carta, M. Croad, R. Malpass-Evans, J. C. Jansen, P. Bernardo, G. Clarizia, K. Friess, M. Lanč, N. B. McKeown, *Adv. Mater.* **2014**, *26*, 3526.

[6] I. Rose, M. Carta, R. Malpass-Evans, M. C. Ferrari, P. Bernardo, G. Clarizia, J. C. Jansen, N. B. McKeown, *ACS Macro Lett.* **2015**, *4*, 912.

[7] M. Carta, M. Croad, J. C. Jansen, P. Bernardo, G. Clarizia, N. B. McKeown, *Polym. Chem.* **2014**, *5*, 5255.

[8] R. Williams, L. A. Burt, E. Esposito, J. C. Jansen, E. Tocci, C. Rizzuto, M. Lanč, M. Carta, N. B. McKeown, *J. Mater. Chem. A* **2018**, *6*, 5661.

[9] X. Ma, B. Ghanem, O. Salines, E. Litwiller, I. Pinnau, *ACS Macro Lett.* **2015**, *4*, 231.

[10] X. Ma, I. Pinnau, *Macromolecules* **2018**, *51*, 1069.

[11] X. Ma, M. A. Abdulhamid, I. Pinnau, *Macromolecules* **2017**, *50*, 5850.

[12] B. Ghanem, N. Alaslai, X. Miao, I. Pinnau, *Polymer* **2016**, *96*, 13.

[13] F. Alghunaimi, B. Ghanem, N. Alaslai, R. Swaidan, E. Litwiller, I. Pinnau, *J. Membr. Sci.* **2015**, *490*, 321.

[14] H. W. H. Lai, F. M. Benedetti, J. M. Ahn, A. M. Robinson, Y. Wang, I. Pinnau, Z. P. Smith, Y. Xia, *Science* **2022**, *375*, 1390.

[15] A. M. Robinson, Y. Xia, *ACS Macro Lett.* **2024**, *13*, 118.

[16] J. Chen, M. Longo, A. Fuoco, E. Esposito, M. Monteleone, B. Comesaña Gándara, J. Carolus Jansen, N. B. McKeown, *Angew. Chem. Int. Ed.* **2023**, *62*, e202215250.

[17] F. E. Rodríguez-González, C. Soto, L. Palacio, A. L. Montero-Alejo, N. Escalona, E. Schott, B. Comesaña-Gándara, C. A. Terraza, A. Tundidor-Camba, *Polym. Chem.* **2023**, *14*, 2363.

[18] R. Swaidan, B. Ghanem, M. Al-Saeedi, E. Litwiller, I. Pinnau, *Macromolecules* **2014**, *47*, 7453.

[19] K. Mizrahi Rodriguez, F. M. Benedetti, N. Roy, A. X. Wu, Z. P. Smith, *J. Mater. Chem. A* **2021**, *9*, 23631.
